# More evidence for prediction model of radiosensitivity

**DOI:** 10.1042/BSR20210034

**Published:** 2021-04-27

**Authors:** Zixuan Du, Xinyan Zhang, Zaixiang Tang

**Affiliations:** 1 Department of Biostatistics, School of Public Health, Medical College of Soochow University, Suzhou 215123, China; 2 Jiangsu Key Laboratory of Preventive and Translational Medicine for Geriatric Diseases, Medical College of Soochow University, Suzhou 215123, China; 3 School of Data Science and Analytics, Kennesaw State University, Kennesaw, GA 30144

**Keywords:** autoencoder, Breast cancer, LASSO Cox regression, radiotherapy sensitivity

## Abstract

With the development of precision medicine, searching for potential biomarkers plays a major role in personalized medicine. Therefore, how to predict radiosensitivity to improve radiotherapy is a burning question. The definition of radiosensitivity is complex. Radiosensitive gene/biomarker can be useful for predicting which patients would benefit from radiotherapy. The discovery of radiosensitivity biomarkers require multiple pieces of evidence. A prediction model of breast cancer radiosensitivity based on six genes was established. We had put forward some supplements on the basis of the present study. We found that there were no differences between high- and low-risk scores in the non-radiotherapy group. Patients who received radiotherapy had a significantly better overall survival than non-radiotherapy patients in the predicted low-risk score patients. Furthermore, there was no difference between radiotherapy group and non-radiotherapy group in the high-risk score group. Those results firmly supported the prediction model of radiosensitivity. In addition, building a radiosensitivity prediction model was systematically discussed. Genes of model could be screened by different methods, such as Cox regression analysis, Lasso Cox regression method, random forest algorithm and other methods. In the future, precision radiotherapy might depend on the combination of multi-omics data and high dimensional image data.

It is well known that radiotherapy plays a significant role in the treatment of cancer as adjuvant therapy. However, heterogeneity of the response to radiotherapy exists among patients. Some patients are more sensitive to radiotherapy while others may not benefit from the radiotherapy. Recently, Chen et al. [1] developed a signature for breast cancer radiosensitivity and this signature shed new light in the treatment of breast cancer.

The definition of radiosensitivity could be different based on various applications scenario. From clinical point of view, we define radiosensitivity for the patients satisfying both of the following criteria: (1) When two groups of patients did not receive radiotherapy, the survival rate of group A (RS group) is not better (equal or worse) than the other group B (NRS group); (2) when both groups of patients received radiotherapy, patients in group A (RS group) obtained significantly more survival benefits than the patients in group B (NRS group). Then patients in group A could be defined as radiosensitive patients (RS group), as shown in Figure 1A. Beyond overall survival, the radiosensitivity could also be defined by other clinical outcomes such as treatment response and tumor size.

**Figure 1:**
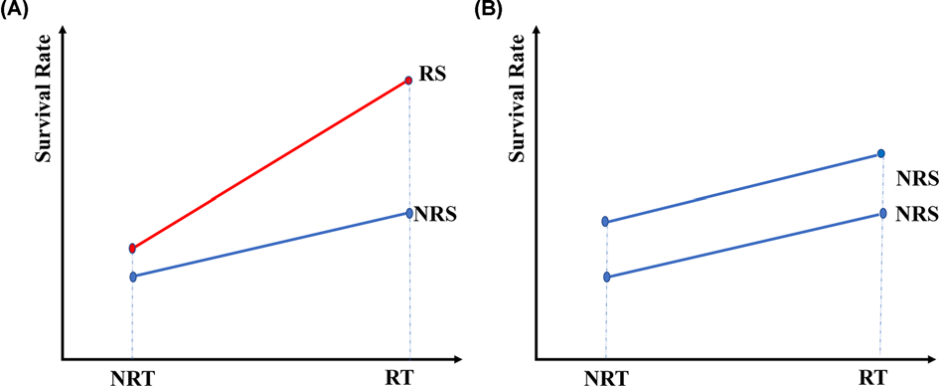
The definition of radiosensitivity and non-radiosensitivity (**A**) Definition of radiosensitivity (**B**) Definition of non-radiosensitivity. Abbreviations: NRS, non-radiosensitivity; NRT, non-radiotherapy; RS, radiosensitivity; RT, radiotherapy.

In summary, to define the radiosensitivity for a group of patients, at least three kinds of test could be given. (1). Survival comparison under non-radiotherapy; (2) Survival comparison under radiotherapy and non-radiotherapy; (3) Survival comparison under radiotherapy. However, these tests cannot be easily applied in clinical settings. Alternatively, it has been known that the genomic background could be one possible cause for the heterogeneity of the response to radiotherapy. Therefore, understanding the radiosensitivity genes as biomarkers will be of utmost importance in optimizing strategies for radiotherapy. Developing gene signature is a practically reliable way to find radiosensitive patients.

Gene signature had been developed for predicting radiosensitive patients in gastric cancer [2] and breast cancer [3]. At the level of a single gene, low expression of *GLIS2* gene might be associated with radiosensitivity in gastric cancer [4] and the low expression of *DDX60 *gene might be associated with radiosensitivity for patients with breast cancer [5]. Radiosensitive biomarker needs to meet four conditions if high expression of one biomarker is associated with radiosensitivity: (1) in high-expression group, the patients in the radiotherapy group had significantly better survival rate than non-radiotherapy group; (2) there was no survival difference between radiotherapy group and non-radiotherapy group in low-expression group; (3) for all radiotherapy patients, the patients in the high-expression group had significantly better survival than patients in low-expression group; (4) for non-radiotherapy patients, there was no survival difference between high- and low-expression group.

Recently, *a six-gene-based signature* established a prediction model for breast cancer radiotherapy sensitivity estimation [1]. The six genes in this signature include *HOXB13, NKX2-2, ADAMTS20*, *LOC284930, ACTL8* and *LOC101928978* [1]. And the researchers constructed a radiotherapy sensitivity prediction model by using those six genes, selected by univariate Cox regression and Lasso Cox regression. The model was further validated International Cancer Genome Consortium (ICGA). The two subgroups, predicted high- and low-risk score subgroups, showed significant survival differences among radiotherapy patients of breast cancer. However, the survival difference might not indicate radiosensitivity. The two groups of patients might be already different in overall survival. They might all benefit from radiotherapy with the same effect size, as shown in Figure 1B. Therefore, it is necessary to compare the survival rate between high- and low-risk score patients who did not receive radiotherapy. We analyzed the overall survival between high- and low-risk scores in the patients who did not receive radiotherapy, as shown in Figure 2A. There were no differences between high- and low-risk scores in the non-radiotherapy group. The comparisons were also performed between radiotherapy and non-radiotherapy groups in high-risk score patients (Figure 2B) and low-risk score patients (Figure 2C). Figure 2B showed that patients who received radiotherapy had a significantly better overall survival than non-radiotherapy patients in the predicted low-risk score patients. Figure 2C showed that there was no difference between the radiotherapy patients and the non-radiotherapy patients in the predicted high-risk score patients. Those results supported the model as an independent signature for predicting radiosensitivity.

**Figure 2:**
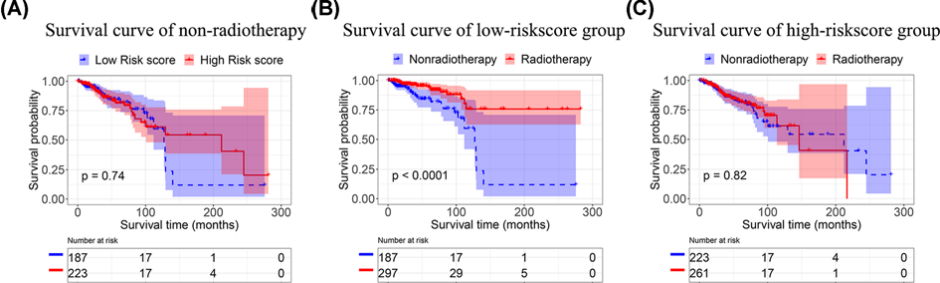
Kaplan-Meier plot of BRCA patients in different group (**A**) Kaplan–Meier plot of BRCA patients in a non-radiotherapy group. (**B**) Kaplan–Meier plot of BRCA patients in the high-risk score group. (**C**) Kaplan–Meier plot of BRCA patients in the low-risk score group.

Building a radiosensitivity prediction model usually has several steps [6]. However, how to construct a radiosensitivity prediction model has not been systematically discussed in past years. Patients who received radiotherapy were usually selected as the modeling population. Patients who did not receive radiotherapy were often ignored. Genes included in the model could be screened by different methods, such as Cox regression analysis, Lasso Cox regression method, random forest algorithm and other methods. Gene selection could also be performed through bioinformatics approach. Recently, Li et al. developed a radiosensitive gene signature by using co-expression and ceRNA network analysis to select genes [7]. The selected genes then were used to build the prediction model. In the independent validation step, the comparison of the survival rate is performed in high/low-risk score in patients who received radiotherapy. In addition, the survival ROC was usually provided to show the prediction power. A nomogram was used to predict the risk score and clinical factors for individual patient.

Establishing a radiosensitivity prediction model would benefit the development of personalized treatment of cancer. The recent emergence of immune checkpoint blockade therapy has spurred an increased interest in the combination of radiotherapy and immunobiology for cancer. The 31 genetic signatures of radiosensitivity were established from four different microarrays by using the NCI-60 cancer cell line [8]. This signature was verified in glioma [9], breast cancer [10] and low-grade glioma [11]. Based on the expression status of programmed death ligand-1 (PD-L1) and the radiosensitivity gene signature, researchers identified a subgroup of PD-L1-high-RR that could benefit from combined immunotherapy with radiotherapy in low-grade glioma. The present study discussed the combination of radiotherapy and immunotherapy in the treatment of cancer from the perspective of PD-L1 [11]. The relationship between genes in the PD-L1 pathway and radiosensitivity in gastric cancer patients was also studied [12].

In summary, the radiosensitivity prediction is helpful for clinical radiotherapy. In the future, precision radiotherapy might depend on the combination of multi-omics data and high-dimensional image data.

## Abbreviation

BRCA: breast cancer
PD-L1: programmed death ligand-1
